# Antibodies raised against conformationally constrained Aβ β‐hairpins: Structural probes and potential immunotherapies

**DOI:** 10.1002/alz70855_099874

**Published:** 2025-12-23

**Authors:** Adam G Kreutzer, Jovanna Plancarte, Jeff Dykstra, James Nowick

**Affiliations:** ^1^ University of California Irvine, Irvine, CA, USA; ^2^ University of California Irvine, IRVINE, CA, USA

## Abstract

**Background:**

Therapeutic strategies aimed at clearing or preventing the accumulation of Aβ have dominated disease‐modifying Alzheimer's disease (AD) drug development for decades, with monoclonal antibody (mAb) immunotherapies at the forefront of this effort. Anti‐Aβ immunotherapies are now showing some promise in the clinic, leading to modest reductions in cognitive decline in AD patients. The only modest reduction in cognitive decline elicited by anti‐Aβ mAbs underscores the need for improved antibodies that lead to a greater reduction in cognitive decline in AD.

**Method:**

mAbs that target disease‐related conformations and assemblies of Aβ offer the promise of improved efficacy. This talk presents the creation and study of monoclonal antibodies designed to target disease‐related conformations adopted by Aβ. These antibodies were raised against peptide antigens that mimic β‐hairpins of Aβ and oligomers formed by the β‐hairpins.

**Result:**

X‐ray crystallography reveals the high‐resolution structures of the antigen binding domains from several mAbs bound to their Aβ targets. These structures reveal atomic‐level details about the Aβ epitopes recognized by the mAbs. Several of the mAbs recognize unique Aβ pathology in AD brain tissue and in 5xFAD mouse brains. The X‐ray crystallographic studies in combination with these immunostaining studies offer clues about the structures and conformations that Aβ adopts in the brain. Immunotherapeutic studies in AD mice will reveal if targeting these epitopes offers therapeutic potential to possibly reduce brain amyloid and lead to cognitive and behavioral improvements.

**Conclusion:**

Monoclonal antibodies generated against conformationally constrained Aβ β‐hairpins and oligomers are new tools for probing the structural landscape of Aβ in the brain. These antibodies offer the promise of new diagnostics and new avenues for AD therapies.

